# Statistics in Medicine

**Published:** 1920-03-13

**Authors:** 


### STATISTICS IN MEDICINE.

We read that the Government is about to be presented with an influentially signed petition urging the immediate necessity for instituting an inquiry into the value of methods so far adopted in collecting and presenting public statistics. There are two main channels of error to which attention must be drawn, namely, that of defective mathematical deduction and that of unavoidable, but nevertheless actual, omission of existing facts. Although this cry for yet another reform to be welded into the country's future organisation does not by any means refer to medical considerations alone, yet so great a part must the public health aspects of a host of national problems play, that we are glad to see our medical contemporaries proceeding to support the urgency of a very necessary measure.

Last week we referred to the potential possibilities at the time the next Census returns are made. There is no doubt that through this channel far more information might be obtained than has been customary in the past.

The fact is that, medically speaking, a great number of people, chiefly of the middle and upper, but also of the lower class, relatively completely escape official recognition in our existing public health returns. Care may thus have been lately lavished upon one class more than another, upon one type of community more than another, and although it is beside the point here to discuss which, if any, have the greater claim to attention, it is certain that with the changing circumstances of the times a high degree of modernisation is required in the majority of our statistical methods.

To those unfamiliar with this aspect of medical work and with vital statistics, let us point out a few fully recognised sources of error, which are at present but partially allowed for. Take the instance of determining the population of any given area. Estimation based on the most recent census returns becomes possibly erroneous both as the period since the last return increases and as the conditions, local emigrations, health, births, etc., fluctuate with changing times such as the last few years. From this error may arise fallacies in the birth-rate, death-rate, in fact all health returns, and so ultimate conclusions may be drawn which, though sound enough from the paper records, are absolutely misleading in every sense. We know that local factors and a variety of methods have from time to time been introduced to rectify these errors, but their success has been far from ideal. In support of our medical departments it may be said that the medical records are probably among the best and most accurate in the country, but all the same they cannot possibly claim perfection. Such abrupt and radical changes of life conditions have occurred in relation to the various classes of people, so many groups and sub-groups have sprung into existence in the general community that without doubt a complete revision of the whole system is required.

Good statistics are not to be mistrusted to the extent that some would have us believe. They are essential to national health, and it is to be hoped that no effort will be spared to bring about an immediate Royal Commission inquiry. There are signs that changes must be made at once; many indeed have already been made; but mostly of a nature calculated to serve for the moment only and for a hasty collection of a few immediate results.

